# Monitoring BRAF and NRAS mutations with cell-free circulating tumor DNA from metastatic melanoma patients

**DOI:** 10.18632/oncotarget.26343

**Published:** 2018-11-16

**Authors:** Elodie Long-Mira, Marius Ilie, Emmanuel Chamorey, Florence Leduff-Blanc, Henri Montaudié, Virginie Tanga, Maryline Allégra, Virginie Lespinet-Fabre, Olivier Bordone, Christelle Bonnetaud, Renaud Schiappa, Catherine Butori, Coraline Bence, Jean-Philippe Lacour, Véronique Hofman, Paul Hofman

**Affiliations:** ^1^ Université Côte d'Azur, CHU Nice, FHU OncoAge, Laboratory of Clinical and Experimental Pathology, Pasteur Hospital, Nice, France; ^2^ Université Côte d'Azur, CNRS, INSERM, IRCAN, FHU OncoAge, Team 4, Nice, France; ^3^ Université Côte d'Azur, CHU Nice, FHU OncoAge, Hospital-Integrated Biobank, Nice, France; ^4^ Antoine Lacassagne Comprehensive Cancer Center, FHU OncoAge, Biostatistics Unit, Nice, France; ^5^ Université Côte d'Azur, CHU Nice, Department of Dermatology, Archet Hospital, Nice, France

**Keywords:** metastatic melanoma, BRAF, NRAS, cfDNA, IDYLLA™

## Abstract

The mutation status of the *BRAF* and *NRAS* genes in tumor tissue is used to select patients with metastatic melanoma for targeted therapy. Cell-free circulating DNA (cfDNA) represents an accessible, non-invasive surrogate sample that could provide a snapshot of the *BRAF* and *NRAS* genotype in these patients.

We investigated the feasibility of the Idylla™ assay for detection of *BRAF* and *NRAS* mutations in cfDNA of 19 patients with metastatic melanoma at baseline and during the course of treatment. The cfDNA genotype obtained with Idylla was compared to the results obtained with matched-tumor tissue and to clinical outcome.

At baseline, 47% of patients harbored a *BRAFV600* mutation in their cfDNA. Two months after targeted treatment the *BRAFV600* mutant cfDNA was undetectable in all patients and 3 were disease-free. Moreover, 15% of patients harbored a *NRAS* mutation that was detected with plasma before treatment. The sensitivity and specificity were 80% and 89% for the *BRAF* status, and 79% and 100% for the *NRAS* status in pretreatment cfDNA compared to results obtained with a tissue test. Due to the small size of the population, no significant correlation was observed between the presence of *BRAF* or *NRAS* mutations in cfDNA and the metastatic tumor load or overall survival.

In conclusion, this study demonstrated that evaluation with the Idylla system of the *BRAF* and *NRAS* mutation status in cfDNA may be a surrogate for determination of the *BRAF* and *NRAS* status in tumor tissue.

## INTRODUCTION

The survival of patients with metastatic melanoma has improved with treatment with BRAF and MEK inhibitors [1] or with immunotherapy such as anti-PD-1 or anti-CTLA-4 antibodies [2, 3]. The mutated *BRAF* oncogene represents a therapeutic target in metastatic melanoma, where a mutated *NRAS* oncogene is a biomarker of poor outcome [4] and resistance to treatment with *BRAF* inhibitors [5]. A prerequisite for safe clinical use of BRAF inhibitors is based on reliable molecular detection of activating *BRAF* mutations in routine clinical practice. Several methods to detect *BRAF* and *NRAS* mutations in formalin-fixed paraffin-embedded (FFPE) samples are currently available in molecular pathology laboratories worldwide. PCR-based techniques require a dedicated infrastructure, which is not always present in pathology laboratories. Moreover, guarantee of reliable and accurate molecular results can be obtained by continuous control of the pre-analytical steps and by a trained technical personnel working ideally in an ISO-accredited laboratory [6, 7]. As only patients whose tumors harbor the “druggable” mutation will benefit from a targeted treatment, there is strong need for reliable, fast, and easy-to-use detection of mutations. Furthermore, a personalized treatment scheme requires monitoring of the tumor's genomic status. Liquid biopsy in metastatic melanoma has emerged as an alternative tool that is complementary to tumor biopsies for detection of “druggable” molecular alterations [8–10]. Many studies have demonstrated that circulating cell-free DNA (cfDNA) represents genetic information from the whole tumor genome and can provide evidence of the clonal evolution and tumor heterogeneity in several types of cancer, including melanoma [11–15]. The detection of the tumoral fraction of cfDNA (ctDNA) is challenging, notably because the relative yield of ctDNA varies significantly, and in some cases less than 1% of the total amount of cfDNA is obtained. The sensitivity and specificity also depends on respecting closely the pre-analytical steps, from sample collection to rapid handling in less than 6 hours [16, 17].

In this study, we evaluated the fully automated ready-to-use Idylla™ PCR-based system for identification of *BRAF V600* and *NRAS* mutations in plasma samples from patients with metastatic melanoma at baseline and during the course of treatment. A comparison with pyrosequencing using matched tissue samples and with next-generation sequencing (NGS) with matched plasma specimens was also performed.

## RESULTS

### Study population

In this monocentric prospective study, samples from 19 patients with stage IV metastatic melanoma collected before and after treatment were assessed for *BRAF* and *NRAS* mutations. The main clinicopathological features of the included patients are highlighted in Table 1. To measure the metastatic tumor burden (TB), the number of metastatic sites was counted before and after therapy [range 1 to 5]. Sites of involvement, in order of frequency, were visceral (15/19; 79%), lymph node (7/19; 37%), subcutaneous (6/19; 32%), brain (5/19; 26%) and bone (2/19; 10%). At the first blood draw, all patients were naïve to treatment. Nine out of 19 (47%) patients received tyrosine kinase inhibitor therapy such as BRAF inhibitors (Zelboraf/Dabrafenib) or combined BRAF and MEK inhibitors (Mekinist); 9/19 (47%) patients received immunotherapy (Ipilimumab or Nivolumab) and one patient was treated with dacarbazine (5%). One patient with bone metastasis also received radiotherapy. 6 out of 19 (32%) patients were clinically disease-free after treatment.

**Table 1:** Main clinicopathological parameters

Demographical and clinical characteristics		Overall
		19 (100%)
**Age at diagnostic**		
(years)	Median	61.63
	Range	43–78
**Gender**	Male	16 (84%)
	Female	3 (16%)
**Histology**	Superficial Spreading	8 (42%)
	Nodular	5 (26%)
	Acral lentiginous	2 (11%)
	Other	4 (21%)
**Breslow (mm)**	Median Range	4.91 (0.35–10)
	Assessed	17 (89%)
**Numbers of metastatic sites before therapy**	1	8 (42%)
	2	5 (26%)
	3	4 (21%)
	4	2 (10%)
	Visceral disease	15 (79%)
	Non visceral disease	4 (21 (%)
**Brain metastasis**	Yes	3 (16%)
	No	16 (82%)
**Treatment**	**Targeted Therapy**	9 (47%)
	BRAF inhibitors	2 (10%)
	Combine MEK and BRAF inhibitors	7 (36%)
	**Immunotherapy**	9 (47%)
	anti-CTLA4	8 (42%)
	anti-PD1	1 (5%)
	**Chemotherapy**	1 (5%)
**LDH**	Assessed	35 (100%)
	Increased	6 (17%)
	Range	504–1826
**Treatment line**	1	19
**Response at M2**	Complete response	3 (16%)
	Partial response	5 (26%)
	Stabilization	5 (26%)
	Progression	6 (31%)
	Death before M2	2 (10%)
**Overall survival (month)**	Median	56.4
	Range	[28.0–NA]
**Mutational status in tumor tissue**	**BRAF**	10 (52%)
	p.V600E	9
	p.V600K	1
	**NRAS**	5 (26%)
	p.Q61K	2
	p.Q61L	1
	p.Q61R	1
	pG12S	1
	**Double Wild-Type**	4 (21%)

### Mutation status of cfDNA evaluated with the Idylla system

At baseline, 9 out of 19 (47%) patients harbored a plasmatic *BRAFV600* mutation with a mean mutant allele frequency of 12.7% [range 0.36%–66.67%]. Two months after treatment the *BRAFV600* mutant cfDNA was undetectable in all patients. For *NRAS* 2 out of 13 (15%) harbored a plasmatic mutation before and after treatment.

### Mutation status in FFPE sample with pyrosequencing and comparison with Idylla in plasma

The pyrosequencing analysis of tumor tissue showed that 10 out of 19 (52%) patients had a *BRAF* mutation, 5 out 19 (26%) harbored a *NRAS* mutation, and 4 out of 19 (21%) patients were WT for both *BRAF* and *NRAS* genes (Table 1).

The agreement at baseline for both approaches was high for the *BRAF* mutation status, with a sensitivity of 80% and a specificity of 89% (κ = 0.69). Three cases out of 19 (16%) had discordant results. Two *BRAFV600E* mutations detected in FFPE samples were not detected in plasma, and one *BRAFV600E* mutation was detected in plasma (0.36% mutant allele frequency) but was not detected on the matched tissue specimen by pyrosequencing, Next Generation Sequencing (NGS) or immunohistochemistry. With regard to the *NRAS* mutation status, 3 FFPE mutations (Q61L; Q61K, G12S) were not detected in plasma. The genotype of matched FFPE samples determined by pyrosequencing showed a concordance rate of 84% (11/13) with a sensitivity of 79% and a specificity of 100%.

### Next generation sequencing with plasma samples and comparison with tissue and Idylla™

**Figure 1:**
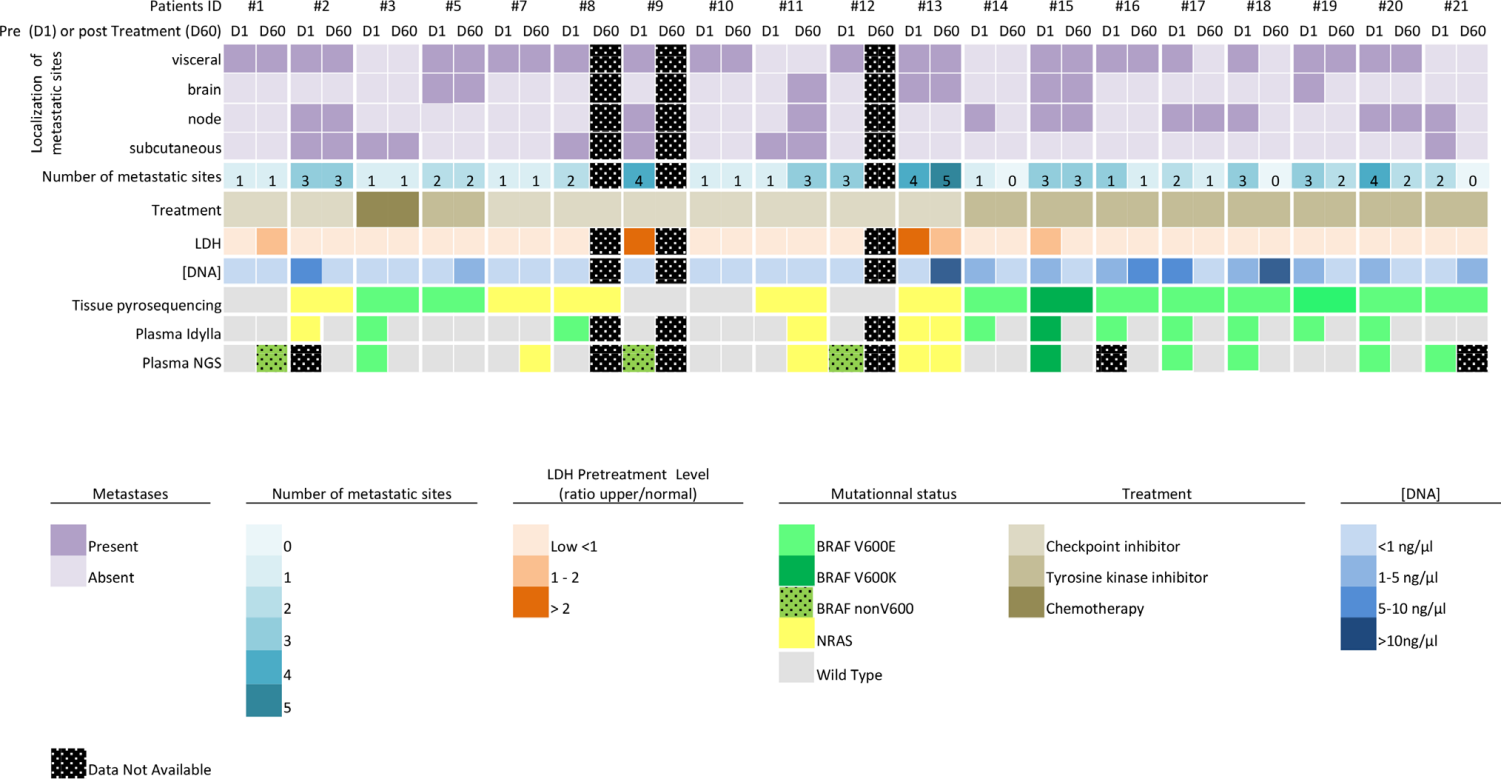
Overview of the results and clinical data

The mutation status was assessed by NGS for all 35 plasma samples included in the study. An adequate amount of DNA was extracted from the plasma samples before and after treatment, however analysis failed for 3 samples. Nine mutations were detected prior to treatment (6 *BRAFV600*, 2 *BRAF nonV600* and 1 *NRASQ61K*) and 4 subsequent to treatment (1 *BRAF nonV600* and 3 *NRAS*) (Figure 1). There was a wide range of the mutant allele fraction (median = 15.1%, range 2.2 to 49%). When compared to the mutation status obtained with FFPE tumor samples, 14 out of 17 (82%) cases showed agreement for *BRAFV600* (sensitivity 67%, and specificity 75%) and 14 out of 17(82%) for *NRAS* (sensitivity 40%, and specificity 100%). Twenty-seven out of 32 (84%) results were in agreement with the findings with Idylla (before and after treatment). When only the plasma *NRAS* and *BRAFV600* mutation spectrum is considered, 5 cases are discordant. 3 *BRAFV*600 (#8, #14, #19) mutations detected with Idylla were absent with NGS, and 1 *BRAFV*600 (#21) and 1 *NRAS (#7)* mutation detected in NGS are not detected with Idylla. For these cases the mutant allelic fraction was low (mean 1.65 range [0.36–3.1]) near by the limit of detection of both methods. Finally, we did not find any KIT gene activating mutations or resistant mutations on PI3K, PTEN, AKT, CDKN2a or JAK2 genes [18, 19], although clonal evolution in melanoma has been described when on therapy [20, 21].

### Mutation status in cfDNA and clinico-pathological characteristics

Among the 3 conflicting cases Idylla *BRAF* prior to treatment, 2 *BRAFV600E* mutations detected in FFPE samples were not detected in plasma. For these patients the time between tissue analysis and plasma is 20 days and 3 months respectively. Both patients (#5 and #21) had a low cfDNA concentration (0.3 ng/μl and 0.8 ng/μl respectively), a normal LDH level, and a low metastatic TB (one brain metastasis and a residual pancreatic metastasis after surgical resection, and one lymph node metastasis and subcutaneous nodule). Both patients were still alive 3 years after the first blood draw when under combined targeted therapy. For the additional *BRAFV600E* detected in plasma (#8) the time between the tissue and the blood sampling is 6 months. When analyzing at the time elapsed between tissue and plasma analysis, we can see that the discrepancy observed in genotype is not linked with the time period in our cohort of naïve treatment patient. After targeted treatment (BRAF inhibitors with or without MEK inhibitors) the *BRAFV600* mutant cfDNA was undetectable in all patients and 3 had no evidence of disease by imagery (CT-scan and TEP-Scan).

For Idylla *NRAS*, 3 patients (patients #7, #8, #11) harboring a *NRAS* mutation in tumor tissue did not show a *NRAS* mutation in the matched plasma samples. These patients also had a low metastatic TB and normal LDL level. Among them, 2 out of 13 (15%) harbored a plasmatic *NRAS* (Q61R and Q61K) mutation detected before treatment. After immunotherapy (anti-PD1 and anti CTLA4), one patient with metastatic progression had a persistent plasmatic *NRAS Q61K* mutation (patient #13), one *NRAS Q61R* mutation was undetectable in a patient with a partial response (patient #2) and a plasmatic *NRAS Q61L* mutation was identified in a patient with disease progression (lymph node and cerebral metastasis - patient #11) (Figure 1).

Using ultra-deep sequencing of cfDNA we discovered 3 new plasmatic *BRAF* mutations (two *L597S #1; #9* and one *K601E #12*; mean allele fraction 26%– range [18–30%]; mean reads 887 – range [301–1998]) that were not investigated with pyrosequencing or Idylla. Two of these mutations were ever present on FFPE matched samples (SSM #12 and nodular #9 melanoma), they were detected retrospectively with the Sanger sequencing method, and one *BRAF L597S* mutation appeared *de novo* in an acral lentiginous melanoma after stabilization on immunotherapy (#1). In the latter case, it seems likely that we have identified an emerging clone under therapy because the time between plasma and tissue analysis did not exceed 2 months, the plasmatic allele frequency was 30% and the percentage of tumor cells in the tissue was 70% (well above the sensitivity threshold). Also case #8 remains inconsistent as neither the *BRAF* nor the *NRAS* mutation was detected with NGS.

### Correlation of the cfDNA mutation status evaluated with Idylla with clinical outcome

*BRAF* and *NRAS* cfDNA mutations were mutually exclusive. The presence of a plasma mutation at baseline did not correlate statistically to OS (*P* = 0.25) and was not related to the level of LDH. Furthermore, when considering only the cohort of *BRAF V600* mutated tumors with FFPE samples, the presence of a *BRAF* cfDNA mutation did not correlate either to OS (*P* = 0.23), or to the objective response rate (*P* = 0.22) or the number and location of metastases (*P* = 0.46).

**Figure 2:**
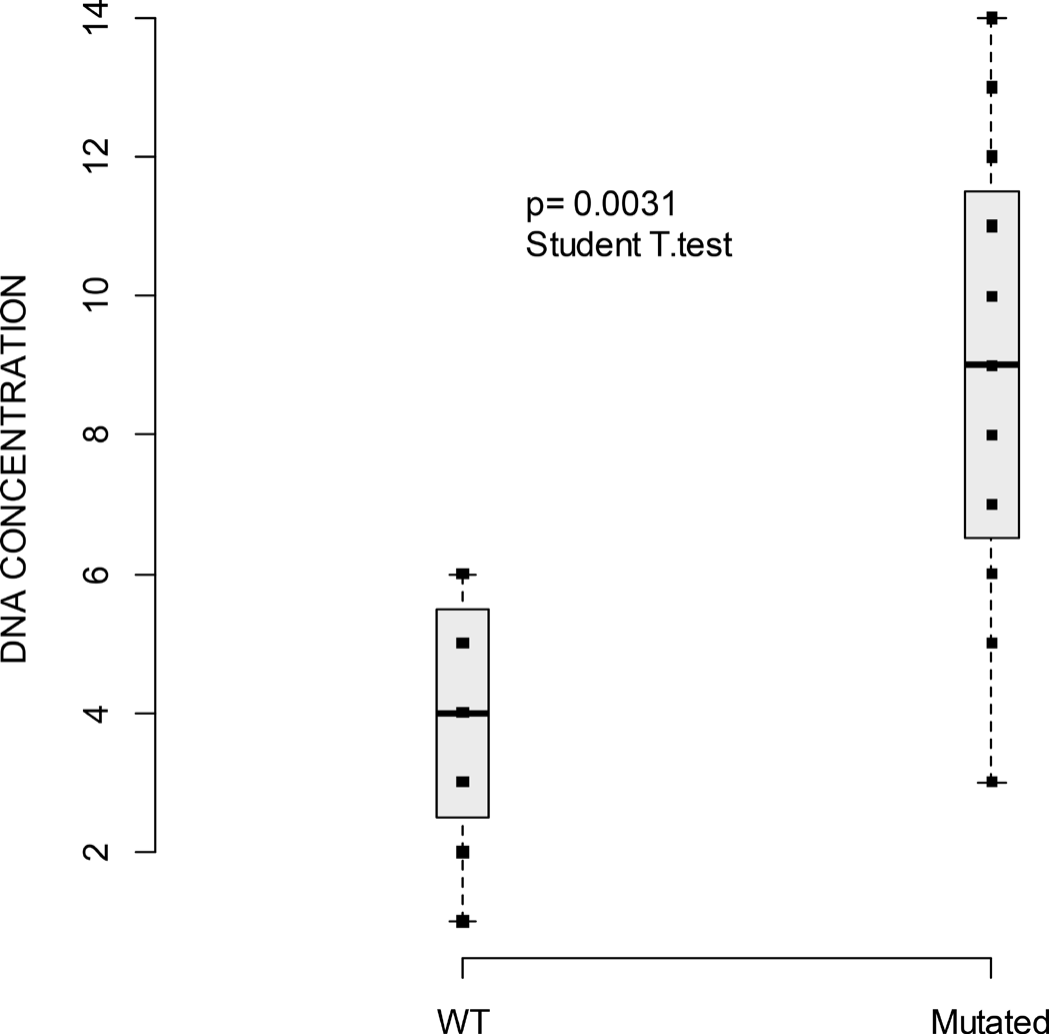
Boxplot of cfDNA concentration (measured with Qubit, ng/μl) and the presence of plasmatic mutations WT versus mutated Horizontal line indicates median = 4 ng/μL (range: 1–6) for WT cfDNA [range 0.6 to 390.0 ng/μL] and median = 9 ng/μL (range: 3–14) for mutated cfDNA. Squares indicate the value of cfDNA. *P*-value of the Student *T*-test indicates a significant difference of cfDNA mean concentration between WT and mutated.

**Figure 3:**
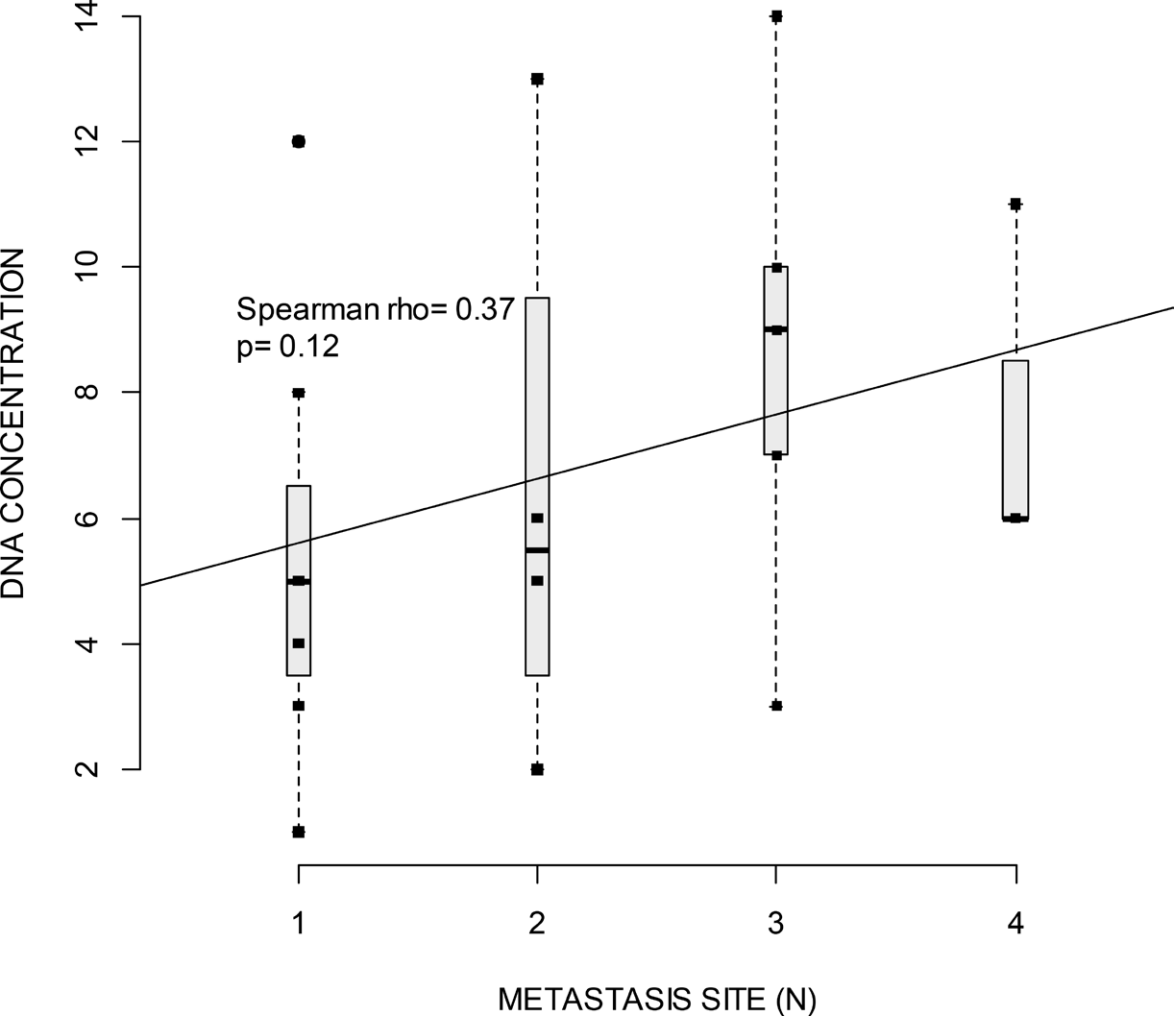
Boxplot of cfDNA concentration (measured with Qubit, ng/μl) and the number of metastatic sites Horizontal lines indicate median for one, two, three or four metastatic sites. Squares indicate value of cfDNA. Spearman rho test indicates correlation between cfDNA and number of metastatic sites. The regression line show a non-significant positive correlation (*p* = 0.12) between the number of metastatic sites and cfDNA concentration.

As we could not assess the yield of cfDNA copies/mL we sought to see if the cfDNA concentration ([cfDNA]) correlated with tumor volume (number and location of metastatic sites), and OS. Measurement of [cfDNA] at baseline was performed for all patients. The mean concentration was 2.77 ng/μL (range 0.27–29 ng/μL). As shown in Figure 2, a correlation between [cfDNA] and the presence of a plasmatic mutation (*P* = 0.0031) was found. The correlation between [cfDNA] and the number or the localization of metastatic sites was not statistically significant. Nevertheless, there was a tendency to correlation between the number of metastatic sites and [cfDNA] (Figure 3). There was a tendency to a higher [cfDNA] for a visceral localization compared to a subcutaneous or brain localization, as previously described in other studies [11, 22, 23]. Moreover, there was no significant correlation between the baseline [cfDNA] or variation before and after treatment, Δ[cfDNA] and OS. The allele mutant fraction of *BRAF* cfDNA was not related to the number of metastatic sites, but there was a tendency towards a higher mutant fraction when a higher [cfDNA] (Spearman *rho* 0.62; *P* = 0.08) was found.

## DISCUSSION

Liquid biopsy is a non-invasive method that allows disease status monitoring during treatment of patients with cancer [24]. While detection of circulating melanoma cells in blood could have a prognostic impact for patients with metastatic melanoma, and despite a few interesting approaches [25, 26], these methods cannot assess the mutation status in clinical routine practice [27]. In contrast, the analysis of cfDNA can play a major role in personalized medicine, especially for cancers that can be treated with targeted therapies [28, 29]. Recently, Valpione *et al*. demonstrated that cfDNA could be a surrogate marker of TB and a prognostic factor for OS in patients with metastatic melanoma [30]. In our study, the number of metastatic sites and the [cfDNA] seemed to be relatively related but we did not find any significant correlation between the [cfDNA] at baseline or after treatment and the TB or OS, as previously described [31]. The lack of correlation between the TB and the [cfDNA] may probably be related to the number of metastatic sites that we added to our analysis to evaluate the TB. Precise evaluation of the TB by imaging is currently lacking in routine practice, probably because it is time consuming, and the RECIST 1.1 criteria that rely on “mono-dimensional” measurements of a maximum of 2 lesions per organ and 5 lesions in total, which is representative of all “involved organs”, does not seem satisfactory to accurately evaluate the relationship between the [cfDNA] and TB [30].

In our study, two *BRAF V600E* mutations detected on a FFPE sample were not detected in plasma in two patients with low TB and low [cfDNA]. This is consistent with recent reports that have demonstrated a lower [cfDNA] level in the presence of subcutaneous [23] or brain metastases [11, 22]. In patients with a cerebral metastasis, the analysis of the cerebrospinal fluid may be an alternative for detection of mutations [32]. Furthermore, in our study, a *BRAF V600* mutation was detected in cfDNA with a small fraction of the mutant allele (0.36%) whereas the matched FFPE tissue sample was WT when using several methods. However, we could not reanalyze the sample as the cfDNA extracted with the Idylla™ device is not available after the experiment. Also the time between blood collection and biopsy was 6 months and even if it seems that it has a low risk impact on the discordant result, we can speculate that a minor emergent clone could have been identified. This would support the hypothesis that the cfDNA profile mirrored the genomic heterogeneity from multiple metastatic sites, which may not be detected with a single tissue biopsy specimen. Moreover, this tumor also harbored a *NRAS Q61K* mutation, which was detected on a subcutaneous biopsy FFPE sample and it seems likely, as previously mentioned for subcutaneous mutations [32], that the *NRAS Q61K* mutation of cfDNA was underrepresented in the blood, as it was not detected with either Idylla or NGS. Lastly, we cannot preclude a false positive result of the Idylla device in front of these elements add to the weak allelic frequency. Thus, we recommend that results with a low allele frequency should be interpreted carefully, be performed in duplicate or on serial analysis, and should always be compared with the total amount of cfDNA.

It is also interesting to monitor patients with a liquid biopsy for detection of mechanisms of resistance to treatment [5, 33, 34]. Although no resistance mutations were detected by NGS in plasma samples, probably due to the short time between initiation of the systemic therapy and the second blood draw, the persistence of the *NRAS* mutations in cfDNA suggests the presence of uncontrolled disease on therapy. Patients with *NRAS* mutations have a worse prognosis with a short OS, highlighting the need to identify these patients for more effective therapy or close surveillance [4].

In our study, the detection at baseline of a *BRAF* mutation in cfDNA was not predictive of response to treatment or OS. While baseline measurement of the *BRAFV600* mutation in plasma with several methods, including Idylla™, has been shown to have prognostic value [10, 31]. In addition, study revealed that the increase in the mutant *BRAF* fraction preceded progression of the disease when detected with imagery giving a median interval of 25 days [35]. We did not find any correlation with OS or ORR with regard to the presence of a cfDNA mutation, the [cfDNA] or the mutant allele fraction. This may be in part due to the small size of the cohort, which also included tumors with *NRAS* mutations that are known to have a poorer prognosis.

Given that the sensitivity of detection of tumoral circulating DNA is low, the results should be interpreted with caution, ideally results should be confirmed with an orthogonal technique but it is sometimes unfeasible in routine manner. That's why it is necessary to validate the limit of detection threshold (LOD) and to define the variation of the background noise in order to avoid any risk of false negatives and positives results. It is also recommended to use an internal validation control with mutations whose detection threshold is low (1 to 2%) close to the LOD. Interestingly, 2 uncommon exon 15 *BRAF* mutations (*L597S*; K501E) were identified by NGS in plasma specimens. These mutations are rare with a reported frequency of 1 to 4% [36, 37]. In our study, 17% (3/17) of patients harbored these mutations (11% *L597S* and 5% *K501E*). When considering only the cohort of double *BRAF* and *NRAS* WT tumors examined by pyrosequencing these mutations were present in 60% (3/5) of patients. Since they are known to be moderately sensitive to BRAF and MEK inhibitors [38], their presence at such a high rate in our population prompted us to perform a more comprehensive sequencing of “pan-negative” melanoma samples.

With the development of new PCR technologies (BEAMing, AS-PCR, ARMS, digital-PCR, NGS) and the improvement of the analytical sensitivity (0.005% to 0.1%) detection of cfDNA, even in early stage malignancies, has demonstrated potential clinical application [11, 39]. The clinical interest of cfDNA detection in breast, colon or lung cancers has been demonstrated for molecular profiling and early detection of resistance; disease monitoring; prognostication or detection of minimal residual disease [22, 40–42]. Recently, the Federal Drug Administration and the European Medicine Agency approved the plasmatic detection of the *EGFR T790M* resistant clone as a companion diagnostic test for second-line treatment of metastatic *EGFR*-mutant non-small cell cancer. Surprisingly, the published results for monitoring *BRAF* or *NRAS* mutant metastatic melanoma have not been adopted in routine clinical oncology. Calapre *et al.* [43] reviewed evidence of the clinical validity of cfDNA as a biomarker for clinical management in metastatic melanoma. CfDNA is an essential source of material for management of metastatic melanoma patients as it allows detection of somatic targeting mutations (BRAF, KIT), copy number variations [23, 44] and the tumor mutation load. In our opinion, we still have to determine the appropriate clinical context of cfDNA analysis.

We compared the three mains methods for cfDNA evaluation of melanoma patients that are available in our laboratory and proposed its clinical application (Table 2). The Idylla™ system offers a fast and easy-to-handle integrated “sample-to-result” approach with results available in less than 2 hours after blood, including plasma, preparation. It would be of particular interest for patients with a new metastatic melanoma, which often evolve rapidly and require urgent identification of the mutation status. Furthermore, the analysis of the *BRAF* and *NRAS* mutation status with cfDNA could be done in a single run, regardless of the mutation detected with the FFPE sample.

**Table 2:** Main technologies available at the Laboratory of Clinical and Experimental Pathology (University Côte d'Azur, Nice, France) and clinical applications

Method	Mutation (Gene)	Analytical sensitivity	Material	Turn around time	Cost per patient tax-free (including plasma extraction)	Proposal of clinical application
**Idylla**™ **assay** (Biocartis, Mechelen, Belgium)	Targeted mutation *BRAF* exon 15 (V600E, E2, K, D, R) *NRAS* exons 12,13, 61 (G12C, G12S, G12D, G12A, G12V, G13D, G13V, G13R, Q61K, Q61L, Q61R, Q61H)	0.1 %	1 ml plasma	90 minutes	115 € for *BRAFV600* & 180 € for both *BRAF* and *NRAS* in one run	Fast identification of targeted mutation when tissue specimen not available / when polymetastatic tumor
**NGS** (Ion PGM, Thermo Fisher Scientific)	Global mutational landscape Copy Number Variation	0.1%	20 ng DNA	3 to 5 days (including data interpretation)	543 € ^*^	Identification of targeted mutation when tissue specimen not available / when polymetastatic tumor Clonal evolution Emergence of resistance
**Crystal digital-PCR** (Stilla, Naica System)	Targeted mutation^*^ BRAF V600E, K, D, R, M *NRAS* exons 12, 13, 61 (G12C, G12S, G12D, G12A, G12V, G13D, G13V, G13R, Q61K, Q61L, Q61R, Q61H) ^*^Note: Need to know the mutational profile of the tumor *One specific primer per reaction*	0.3 copies DNA / μl	10 ng DNA	24 hours	Approx. 114 € for BRAFV600E ^*^depends on the number of primer tested	Longitudinal monitoring of residual disease, response to therapy, relapse

When we compared NGS, which requires a complex workflow and a turnaround time of several days due to manual handling, to the Idylla™ system, the latter seems more effective in routine practice as it could also be implemented in a standard pathology laboratory. NGS has a more restricted indication, mainly during the course of the disease, to identify new biomarkers of resistance and potential therapeutic targets under the cover of a molecular medical and paramedical staff. Finally, digital-PCR allows precise quantification of tumor DNA and monitoring of response, especially in the era of checkpoint inhibitor therapy, with the need to distinguish between false “pseudo” progression and true progression and to oversee long-term responders. Thus, digital-PCR allows the study of the kinetics and variation of cfDNA over a longitudinal period with serial blood analyses.

The information obtained with cfDNA (e.g. somatic druggable mutations, copy number variation or the tumor mutation load) should not be ignored for the management of melanoma patients because it can provide a real-time and global assessment of the patient's status [44, 45]. This analysis should be associated with imagery to reach the best practice [23] for patient care. Large prospective clinical studies are needed to evaluate the medical impact of cfDNA-guided decisions [43].

Our study holds a number of limitations, including the small size of the population and the limited number of longitudinal blood analyses. To our knowledge, we describe here, the first prospective study using the Idylla ctNRAS Assay and a NGS panel for cfDNA analysis in advanced melanoma patients before and after initiation of therapy. CfDNA is particularly interesting in polymetastatic patients as it could reflect genomic tumor heterogeneity.

In conclusion, we demonstrated that the detection of the *BRAF* and *NRAS* cfDNA status with the Idylla™ assay is feasible in a routine manner in a pathology laboratory, before and after systemic treatment of patients with metastatic melanoma. The assay reached acceptable concordance when compared with standard molecular analyses with matched tumor tissue. This approach could offer a good alternative to a surgical biopsy in fragile patients or patients with inaccessible metastatic sites to assess “druggable” molecular alterations.

## MATERIALS AND METHODS

### Patients and samples

Twenty-one consecutive treatment naïve patients with metastatic melanoma followed at the Department of Dermatology (Archet Hospital, Nice, France) were screened for the purpose of the study. 2 patients (#4; #6) were immediately excluded because the inclusion criteria were not met (e.g. both had choroidal melanoma, out of the scope of the study). Patients gave their informed consent and the study was conducted according to the Helsinki guidelines. All patients had a blood test at baseline and 16/19 (84%) had a blood test at their first evaluation two to three months (mean 81 days [range 63–94 days]) after initiation of treatment to evaluate the BRAF and *NRAS* status of cfDNA. The *BRAF* and *NRAS* status of FFPE blocks of corresponding tumors were available for all patients. Tumor specimens were selected by senior pathologists (EL, MI, CB, VH). The percentage of tumor cells (range 30%–80%, mean 65%) was evaluated independently by these pathologists, according to the French association for quality assurance in pathology (AFAQAP) procedures [46]. When several tissue samples were available the most recent resected tumor was selected. The time between tissue analysis and plasma collection ranged from less than 1 month to 38 months (mean 9.5 months). The main clinicopathological parameters are summarized in Table 1. Correlation of the results with the OS was evaluated. The therapeutic response was evaluated with RECIST1.1 criteria (Response Evaluation Criteria in Solid Tumors) on targeted lesions on CT imagery, the same day of the blood draw or within less than one month. The tumor burden (TB) was evaluated by addition of the number of metastatic sites (Table 1).

### Circulating free DNA

Blood samples (10 mL) were collected in EDTA tubes and processed within 4 hours. The plasma was obtained by two rounds of centrifugation (10 minutes, 2000 g at 4°C) aliquoted into 1mL tubes and stored at −80°C until use. For the Idylla assay, DNA was extracted with the specific cartridge and on-board reagents, whereas for Ion Torrent PGM sequencing the DNA was extracted with the Kit QIAamp circulating Nucleic Acid (Qiagen, Hilden, Germany). The cell free DNA concentration was measured with the Qubit Fluorometric Quantitation (ThermoFisher Scientific) double strand (ds) DNA assay kit.

### The BRAF and NRAS mutation status evaluated with Idylla

The Idylla™ platform (Biocartis, Mechelen, Belgium) is a fully cartridge-based automated platform processing with all reagents on-board [31]. The sensitivity was high, down to 0.1% mutant allele, according to the manufacturer's instructions. The plasma was tested for *BRAF* and *NRAS* before and after treatment (when available) with the Idylla™ ctNRAS-BRAF Mutation Test (prototype). For the cfDNA assay, 1 ml of plasma was directly placed into the cartridge. After a 90-minute run and less than 1-minute hands-on time, all steps were automatically performed inside the cartridge and final reports were directly available on the console after an automatic on-board post-PCR curve analysis. As the real time PCR using allele specific primers separated into two chambers the results were given as “V600E/E2/D Mutation” or “V600K/R/M Mutation” or Wild Type. *NRAS* mutations were detected in the same way in codons 12, 13, 59, 61, 117, 146 (according to manufacturer's guidelines). For the *BRAF* Assay, the *BRAF* mutant fraction was established on calculation of the ΔCq (CqMut-CqWT), but this was not possible for *NRAS* (Cq not available).

### BRAF and NRAS Pyrosequencing

DNA extraction was performed with the QiAmp DNA FFPE Tissue Kit (Qiagen, Hilden, Germany) and pyrosequencing analysis was performed as previously described with the therascreen *BRAF* Pyro Kit (Qiagen) and the therascreen *NRAS* Pyro Kit (Qiagen) [47–49]. The platform of the LCEP, (Nice, France) has been accredited for molecular testing by pyrosequencing of exons 11 and 15 of the *BRAF* gene and for codon 12, 13 and 61 of the *NRAS* gene. The laboratory holds full COFRAC (“Comité Français d'Accréditation”) accreditation for these assays, according to the ISO 15189 norm (http://www.cofrac.fr). The analytical sensitivity of the pyrosequencing method was considered to be 5% according to the manufacturer (Qiagen). Pyrogram profile analyses and interpretation of the results were done blindly with PyromarkQ24 (Qiagen) by five independent assessors (EL, MI, VL, VH OB).

### Next generation sequencing

Sequencing libraries were prepared from cfDNA using the Ion AmpliSeq™ Cancer Hotpost Panel v2, encompassing for of a broad set of 50 cancer-relevant genes (list of targeted exonic, intronic and regulatory regions available on https://www.ampliseq.com). According to manufacturer's protocol; 10 ng of DNA for each sample was used as input for library preparation with the Ion AmpliSeqTM Library kit 2.0 (Thermo Fisher). The pooled barcoded libraries were processes on the Ion Chef™ System. The FASTQs sequencing data were aligned to the human genome using the Torrent Suit server. Analysis was then performed with two software VariantCaller (version 2.4.1) and Nextgene (2.4.1-UG001) by two pathologists and three bioinformaticians (VL, OB, CR).

### Statistical analysis

Qualitative data are presented as absolute and relative frequencies and are compared according to the Chi2 test or Fisher exact test when necessary. Quantitative data are presented as mean, standard deviation, median and range and compared using the Student's *t*-test or Wilcoxon test. Survival data are defined between the date of the metastasis diagnostic and the event onset date. Survival curves are compared with the Log-Rank test. Median follow-up was assessed using the reversed Kaplan–Meier method. Concordance between the baseline cfDNA *BRAF* and *NRAS* mutation status and the results in the reference tissue were assessed using the Cohen's kappa coefficient (κ), if indicated, we also evaluated sensitivity and specificity. Correlation between cfDNA levels, the *BRAF* mutant fraction and the baseline tumor burden were assessed using Spearman correlation coefficients. All statistical analyses were two-sided and a *p*-value of less than 0.05 was considered as statistically significant. All calculations were made with R.3.4.3 software.
